# Ferroptosis: a new regulatory mechanism in neuropathic pain

**DOI:** 10.3389/fnagi.2023.1206851

**Published:** 2023-09-22

**Authors:** Lu Li, Lingling Guo, Rui Gao, Mengwen Yao, Xinyu Qu, Guangwei Sun, Qi Fu, Cuntao Hu, Guang Han

**Affiliations:** ^1^Department of Anesthesiology, Shengjing Hospital of China Medical University, Shenyang, China; ^2^Department of Anesthesiology, The Fourth Affiliated Hospital of China Medical University, Shenyang, China

**Keywords:** ferroptosis, neuropathic pain (NP), neuroinflammation, neurodegenerative diseases, reactive oxygen species (ROS)

## Abstract

Neuropathic pain (NP) is pain caused by damage to the somatosensory system. It is a common progressive neurodegenerative disease that usually presents with clinical features such as spontaneous pain, touch-evoked pain, nociceptive hyperalgesia, and sensory abnormalities. Due to the complexity of the mechanism, NP often persists. In addition to the traditionally recognized mechanisms of peripheral nerve damage and central sensitization, excessive iron accumulation, oxidative stress, neuronal inflammation, and lipid peroxidation damage are distinctive features of NP in pathophysiology. However, the mechanisms linking these pathological features to NP are not fully understood. The complexity of the pathogenesis of NP greatly limits the development of therapeutic approaches for NP. Ferroptosis is a novel form of cell death discovered in recent years, in which cell death is usually accompanied by massive iron accumulation and lipid peroxidation. Ferroptosis-inducing factors can affect glutathione peroxidase directly or indirectly through different pathways, leading to decreased antioxidant capacity and accumulation of lipid reactive oxygen species (ROS) in cells, ultimately leading to oxidative cell death. It has been shown that ferroptosis is closely related to the pathophysiological process of many neurological disorders such as NP. Possible mechanisms involved are changes in intracellular iron ion levels, alteration of glutamate excitability, and the onset of oxidative stress. However, the functional changes and specific molecular mechanisms of ferroptosis during this process still need to be further explored. How to intervene in the development of NP by regulating cellular ferroptosis has become a hot issue in etiological research and treatment. In this review, we systematically summarize the recent progress of ferroptosis research in NP, to provide a reference for further understanding of its pathogenesis and propose new targets for treatment.

## 1. Introduction

Neuropathic pain (NP) is a pain syndrome resulting from injury to the peripheral or central nervous system (Chen et al., 2011). It is characterized by mechanical abnormal pain and thermal nociceptive hypersensitivity (Wong et al., 2022). Neuropathic pain is experienced by 6.9–10% of the world’s population (Pickering et al., 2017). Due to the complex pathogenesis of NP, effective treatments are lacking. It has become a public health problem that seriously affects people’s quality of life (Wu, 2016). It imposes a heavy burden on families and society (Liang et al., 2019). Existing clinical treatments such as opioids and Nonsteroidal Anti-inflammatory Drugs (NSAIDs) cannot effectively relieve the clinical symptoms of most NP patients and they often cause a variety of adverse effects (Shinozaki et al., 2015; Vincenzi et al., 2022). In addition, the pathogenesis of NP is not fully understood, which greatly limits the development of therapeutic approaches for NP. Previous studies have shown that NP is provoked and maintained by mediators released from neurons and glial cells that induce neuronal sensitization in the peripheral and central nervous systems (Scholz and Woolf, 2007). In addition to neurons, NP involves many non-neuronal cells, including inflammatory cells, immune cells, and glial cells (Ji et al., 2013; Malcangio, 2019). It has also been shown that immune responses occurring at damaged sites can activate the circulating immune cells in local tissues (McCauley and Baloh, 2019). This process plays an important role in neuronal sensitization in both the periphery and the center. Despite recent advances in the understanding, diagnosis, and treatment of NP, the goal of effective reliable, and durable treatment remains elusive (Liem et al., 2016). It plays a significant negative impact on patients’ somatic function and quality of life (Obara et al., 2020).

It has been shown that reactive oxygen species (ROS) and oxidative stress are involved in the development of NP. Under normal physiological conditions, intracellular antioxidants are in dynamic balance with intracellular ROS (Bi et al., 2022). When mitochondrial damage occurs, ROS production is significantly higher compared to normal physiological levels. And endogenous antioxidants are severely depleted, resulting in a disruption of the balance, leading to intracellular oxidative stress (Bi et al., 2022). Reactive oxygen species scavengers can alleviate NP. Some researchers found that 2-methyl-N-phenylmethylene-2-propanamine N-oxide (PBN) is a potent ROS scavenger (Kim H. K. et al., 2010). It is effective in relieving NP especially when administered intrathecally. Although ROS is involved in the spinal sensitization mechanism of NP, the exact mechanism is not known. The current study concluded that high levels of ROS are associated with the cell death signaling pathways (Zaidieh et al., 2019). Reactive oxygen species can regulate ferroptosis through various pathways. Excessive iron content can cause the accumulation of ROS, which in turn induces lipid peroxidation and ferroptosis (Huang et al., 2021). On the other hand, ferroptosis can also induce lipid peroxidation through intracellular production of lipid ROS via the Fenton reaction (Cheng et al., 2021).The ferroptosis signaling pathway is also involved in the development of NP (Wang et al., 2021).

Cell death modalities are mainly divided into non-programmed and programmed death. Non-programmed cell death is cell death that is directly caused by physical and chemical factors and cannot be regulated. Programmed cell death, on the other hand, is modifiable (Bi et al., 2022). Ferroptosis as new programmed cell death is morphologically manifested mainly by the rupture of the outer mitochondrial membrane, the increase in the density of the inner mitochondrial membrane, and the reduction or disappearance of the mitochondrial cristae (Xie et al., 2016). In terms of metabolic alterations, it is mainly manifested by metabolic changes such as iron deposition, intracellular reactive oxygen species alterations, and lipid peroxidation damage of cell membrane (Zhang N. et al., 2022). Currently, the detection of ferroptosis in cells is also based on ferroptosis morphology and metabolic alterations. Transmission electron microscopy can be used to detect intracellular mitochondrial morphology and changes in tissue iron content (Wang et al., 2020). We often use the changes of intracellular oxidative stress-related indicators such as ROS, malondialdehyde (MDA), and L-Glutathione (GSH) to determine whether ferroptosis occurs in cells (Shuhua et al., 2012). In one study, it was pointed out that ferroptosis can be activated by small molecules such as erastin (Dixon et al., 2012). And the key to its occurrence is the inhibition of recombinant glutathione peroxidase 4(GPX4) activity (Yang et al., 2014). After the reduction of GPX4 activity, the production of intracellular ROS increases and ROS accumulation occurs, which aggravates the occurrence of intracellular lipid peroxidation, and induces the occurrence of ferroptosis (Kim et al., 2021). Therefore, GPX4 is a key protein in ferroptosis and can be used as a signature protein for ferroptosis detection. In addition, other researchers have found that ferrostatin (Fer-1), a specific inhibitor of ferroptosis, significantly inhibits oxidative stress and inhibits the accumulation of ROS in rotenone-treated dopaminergic neuroblastoma cells (SH-SY5Y), thus playing a neuroprotective role (Kabiraj et al., 2015). The investigators also induced ferroptosis in cells of the hippocampal region of rats by high concentrations of glutamate, which could be attenuated by the ferroptosis inhibitor Fer-1 (Magtanong and Dixon, 2018). Currently, neurodegenerative diseases such as Parkinson’s disease (PD) and Alzheimer’s disease (AD) are popular areas of ferroptosis research (Wang et al., 2022). A related study has confirmed that pain levels in rats after nerve injury also vary with the occurrence of ferroptosis.

## 2. The role of reactive oxygen species in neuropathic pain

Neuropathic pain is a chronic pain that results directly from trauma or disease involving the somatosensory system, including peripheral and central sensitization after nerve injury (Treede et al., 2008). It has been shown that NP is associated with enhanced ROS-related oxidative activity (Salvemini et al., 2011). Reactive oxygen species plays an important role in the process of NP. It has been demonstrated that intracellular ROS can activate many signaling pathways and induce cell death (Zhao et al., 2017; Holze et al., 2018). Induction of neuronal death in the dorsal horn of the spinal cord may be an important mechanism for the involvement of ROS in NP (Woller and Hook, 2013). Recent studies have found that ROS scavengers such as PBN, vitamin E, and edaravone can alleviate the behavioral manifestations in animal models of NP (Mao et al., 2009; Kim H. K. et al., 2010). The effect of intrathecal injection of ROS scavengers was the most pronounced.

In a chronic constriction injury (CCI) model of NP in the sciatic nerve, researchers found that XO activity, which was often used to indirectly represent the level of ROS, was approximately four times higher on the operated side of the sciatic nerve than that on the sham-operated control and non-operated side (Khalil et al., 1999). Some researchers also found that MDA levels were higher in the operated side of the sciatic nerve in CCI rats than in sham-operated controls (Naik et al., 2006). Since ROS converts cell membrane phospholipid A to MDA through lipid peroxidation, MDA can also reflect the degree of lipid peroxidation. In addition, if ROS scavenging ability is weakened or ROS-related oxidative activity is enhanced, it also aggravates NP. Superoxide dismutase (SOD) can penetrate the cell membrane, disproportionate superoxide anions, form H_2_O_2_ and O_2_, and play a role in scavenging ROS (Zhang et al., 2023). Tempol is a SOD analog that effectively neutralizes ROS. A previous study has described that CCI rats with an intraperitoneal injection of Tempol had a significantly lower thermal pain threshold (Tal, 1996). It demonstrated that ROS scavenging alleviated NP. Reduced glutathione is the main small molecular weight free radical scavenger in the cytoplasm, which can reflect the antioxidant activity of the body (Mohamed et al., 2020). One study found reduced levels of glutathione in the sciatic nerve on the surgical side of CCI rats. In addition, the free radical trapping agents PBN and edaravone can effectively trap oxygen radicals, generate stable free radical adducts, attenuate ROS activity, and improve the mechanical pain sensitivity in spinal nerve ligation (SNL) rats. It has been reported in the literature that ROS is involved not only in the onset of NP but also in the maintenance of NP. And PBN not only significantly increased the mechanical pain threshold but also delayed the onset of neuropathic pain behavior in SNL rats after the formation of NP. The duration of analgesic effect of PBN greatly exceeds its half-life. This may be due to the need to scavenge reactive oxygen species to recover their injury threshold and thus delay the onset of pain. One interesting study found that ROS clearance relieved pain behavior in SNL model rats without affecting the ectopic firing of neurons in the L5 dorsal root ganglion. This may be because ROS acts in the spinal cord, whereas the ectopic discharges developed after peripheral nerve injury originate from the injured sites and the dorsal root ganglion (DRG) neurons (Sun et al., 2005). Reactive oxygen species scavengers can cross the blood–brain barrier and enter the spinal cord, reducing central sensitization. Therefore, the relationship between ROS and afferent nerve ectopic discharges needs to be further investigated. In conclusion, the effect of ROS on NP is particularly important.

According to previous literature, the main sites of action of ROS mediating NP are in the spinal cord, neurons, and glial cells (Kim D. et al., 2010; Yowtak et al., 2011). One study found that intrathecal injection of the PBN alleviated mechanical nociceptive hyperalgesia in SNL mice. It was demonstrated that ROS may mediate NP at the spinal cord level. In addition, one study found an increase in MDA in the DRG of SNL rats after the administration of ROS agonists, confirming that ROS can also act on neurons. And inflammatory factors are upregulated to activate spinal microglia after nerve injury in L5 spinal nerve transection (SNT) model mice, which are activated by nicotinamide adenine dinucleotide phosphate oxidase 2(NOX2) to produce ROS (Kim D. et al., 2010).

Current studies have found that ROS affects NP in three main ways: (1) ROS affects NP by mediating mitochondrial dysfunction. Mitochondrial division and fusion are dynamic processes (Chandra et al., 2017). When peripheral nerve injury occurs, the damaged mitochondria produce large amounts of ROS, and the accumulation of ROS in the cell disturbs the dynamic balance of mitochondria, induces mitochondrial division, causes peripheral nerve injury to worsen, and induces NP (Sun et al., 2022). Mitochondrial dynamin-related protein 1 (Drp1) is a guanosine triphosphatase (GTPase) that catalyzes mitochondrial division (Chan, 2012). One study found that intrathecal injection of Drp1 antisense oligonucleotides inhibited Drp1 expression and effectively relieved NP (Ferrari et al., 2011). (2) Reactive oxygen species affects NP by mediating central sensitization, which is an increase in sensitivity to peripheral afferents in spinal pain-associated conductance neurons after NP develops. Ectopic firing into the spinal cord after afferent nerve injury leads to a mitochondrial “respiratory burst.” Then an increase in intracellular calcium ions results in increased ROS production, which may be one of the mechanisms underlying central sensitization to NP. Local anesthetics (LAs) such as lidocaine are used to relieve NP by blocking injured nerve afferents (Song et al., 2011). One study found that ROS decreased inhibitory transmission. Minute excitatory postsynaptic potentials and inhibitory postsynaptic potentials of spinal cord dorsal horn neurons recorded with membrane clamp revealed that ROS did not affect the amplitude and frequency of excitatory postsynaptic potentials but decreased the frequency of inhibitory postsynaptic potentials. Reactive oxygen species was involved in central sensitization caused by decreased presynaptic inhibitory transmission and decreased synaptic γ-aminobutyric acid(GABA) release in the spinal cord. Furthermore, central sensitization of NP following ROS activation is associated with altered synaptic plasticity induced by AMPA receptors (Li et al., 2020b). In addition, α-aminohydroxymethoxazole propionic acid (AMPA) receptors are ionotropic glutamate receptors (Jitsuki et al., 2011). Regulation of AMPA receptor transport and activity is a key mechanism of regulating synaptic plasticity and potentiation (Li et al., 2014). It has been found that ROS-activated kinase activates AMPA receptor phosphorylation in the dorsal horn of the spinal cord, which in turn increases the permeability of AMPA receptors to calcium ions on the cell surface (Woller and Hook, 2013). The ROS scavengers PBN and tempol also alleviate neuropathic pain by reversing AMPA receptor phosphorylation activation (Lee et al., 2012). (3) Reactive oxygen species affects NP through signaling pathways. Activation of the signaling pathway MAPK can increase gene expression and modify expressed proteins to maintain plasticity in dorsal root ganglia and spinal dorsal horn neurons. It had been reported that ROS activated extracellular regulated protein kinases (ERK) and p38 phosphorylation in spinal microglia in spinal cord injury (SCI) rats. And microglia production of Prostaglandin E2(PGE2) increased through ERK-dependent signaling pathways, leading to NP. Removal of ROS not only relieves NP but also reduces the phosphorylation levels of ERK and p38 (Kim et al., 2004; Huang et al., 2018; Fan et al., 2020).

## 3. Metabolic mechanisms of ferroptosis

**Figure 1:**
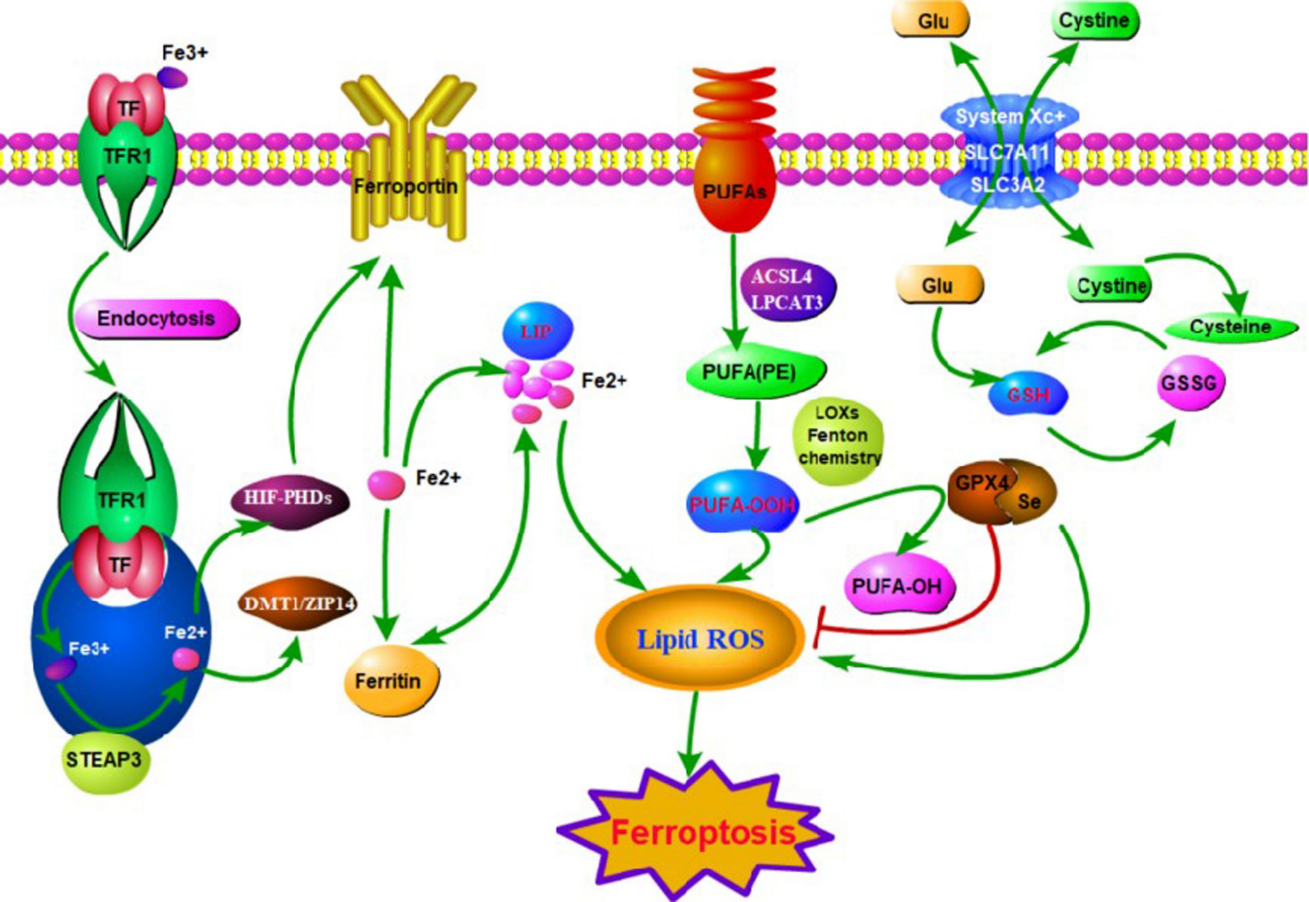
The main mechanisms of ferroptosis in neuropathic pain (NP). Ferroptosis can be initiated through transferrin endocytosis linked to transferrin receptor 1 (TFR1). After endocytosis, ferric iron is released and reduced to ferrous iron (Fe^2+^). Fe^2+^ can be stored in ferritin or remain as a labile iron pool (LIP). The LIP generates lipid peroxidation through fenton reaction. Lipid peroxidation can also occur via enzyme. However, it is necessary for the free polyunsaturated fatty acids (PUFAs) to be esterified as membrane PUFA by the enzymes ACSL4 and LPCAT3. Deoxygenation generates PUFA-OOH, which reacts with other membrane lipids, forming pores in the lipid bilayer, destabilizing it and then rupturing the membrane. Ferroptosis is inhibited by GPX4, which converts PUFA-OOH to alcohol and water. This reaction occurs through the use of glutathione (GSH) as a substrate. GSH synthesis occurs via the entry of cystine into the cell by system xc− Glutathione peroxidase 4 (GPX4) is the major endogenous mechanism to suppress lipid peroxidation. High extracellular concentrations of glutamate inhibit system xc−, which imports cystine by exchanging intracellular glutamate for extracellular cystine. Cystine is subsequently converted to cysteine, which generates glutathione (GSH), a cofactor for GPX4.

**Figure 2:**
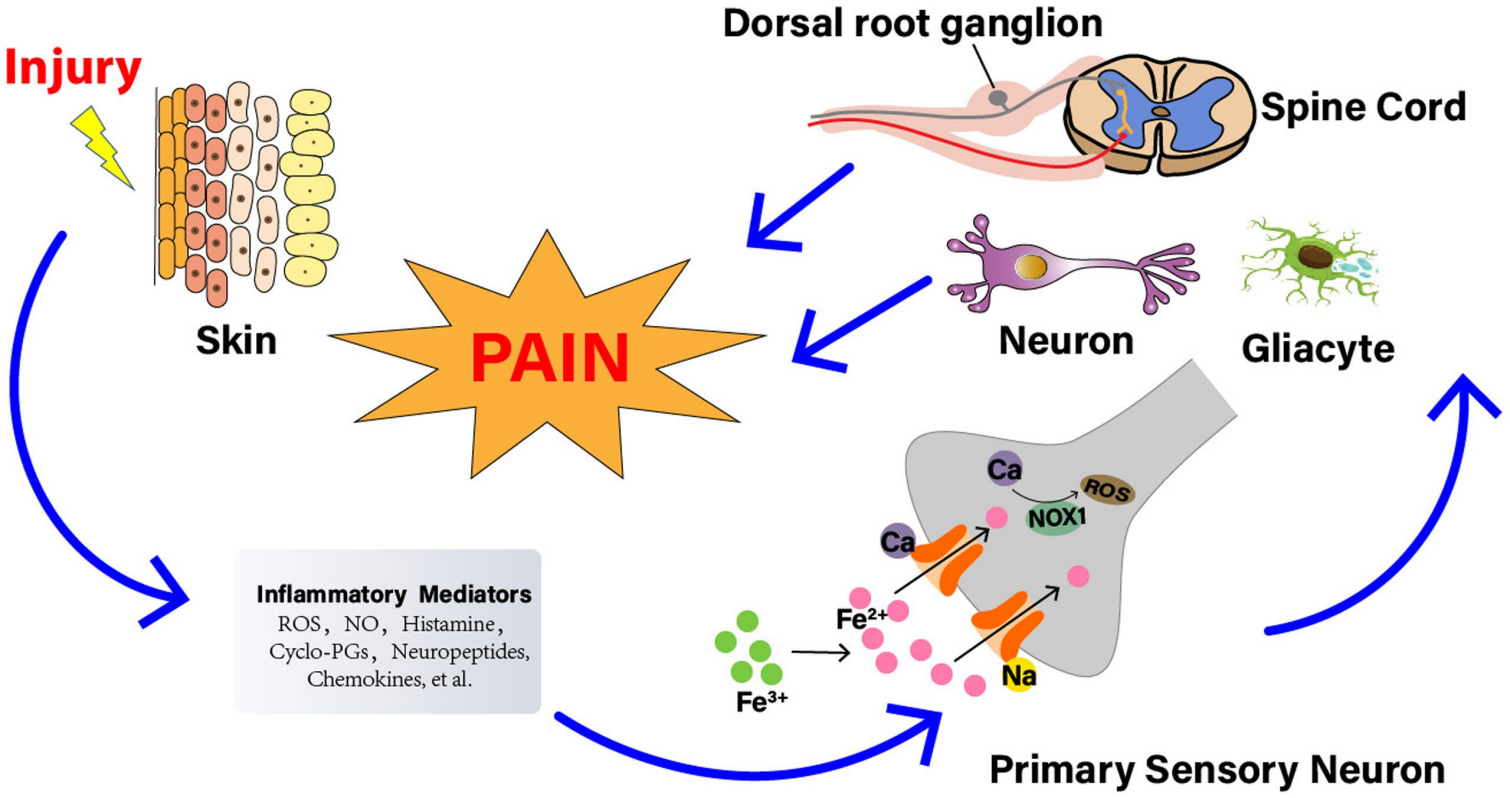
The relationship between reactive oxygen species (ROS) and NP. Nerve injury generates a series of inflammatory agents, including ROS, nitrogen (nitric oxide), bradykinin, prostaglandins, histamine, kinins, cytokines, neuropeptides, and chemokines. Some agents, such as ROS, cyclopentenone-prostaglandins (cyclo-PGs), and nitric oxide, directly gate the channel, evoking a calcium (Ca^2+^)-dependent, NADPH oxidase 1 (NOX1)-mediated amplification of hydrogen peroxide (H_2_O_2_) release, eventually leading to signal mechanical allodynia. The injured nerve trunk releases proinflammatory chemokines, which recruit activated the signal pathways involved with neuronal cells, DRG, glial cells, and spinal cord. And the activation of these pathways contributes to the generation of pain, especially nociception and allodynia.

Many molecular and metabolic pathways are involved in the regulation of ferroptosis (Figure 1). The most extensive studies on cysteine reduction and GPX4 reduction-induced lipid peroxidation have been conducted. Depletion of cysteine decreases GSH synthesis. Depletion of GPX4 blocks the chemical reactions involved in GSH. Cysteine-glutamate anti-transport protein (xc^−^ system) promotes GSH synthesis by increasing cysteine uptake. And molecules such as Activating Transcription Factor 3(ATF3) and Glu promote ferroptosis by inhibiting this complex. Notably, epigenetic regulation such as histone modifications and microRNA-mediated gene silencing also play a crucial role in ferroptosis. In conclusion, the regulation of iron metabolism is an intricate network. Here are some of the important mechanisms involved (Figure 2).

### 3.1. NRF2 /xc^−^ system/GPX4 axis

Nuclear factor erythroid 2-related factor 2 (NRF2) is a key transcription factor in the cellular antioxidant response (Owona and Schluesener, 2015). And a major iron signaling molecule in the nucleus that plays a protective role against oxidative stress damage in disease. Under normal physiological conditions, NRF2 binds to Kelsey-like ECH-associated protein 1 (Keap1) in the cytoplasm and is degraded via the ubiquitin-proteasome pathway (Itoh et al., 1999). During oxidative stress, NRF2 monitors the cellular response to oxidative stress by mediating detoxification through dissociation from the cytoplasmic inhibitor Keap1, which then translocates to the nucleus and activates the NRF2 transcriptional gene to provide cellular protection against oxidative stress (Hayes and McMahon, 2009). This activation of NRF2 reduced iron intake, limited ROS production, and strengthened the antioxidant capacity of the cells.

**Figure 3:**
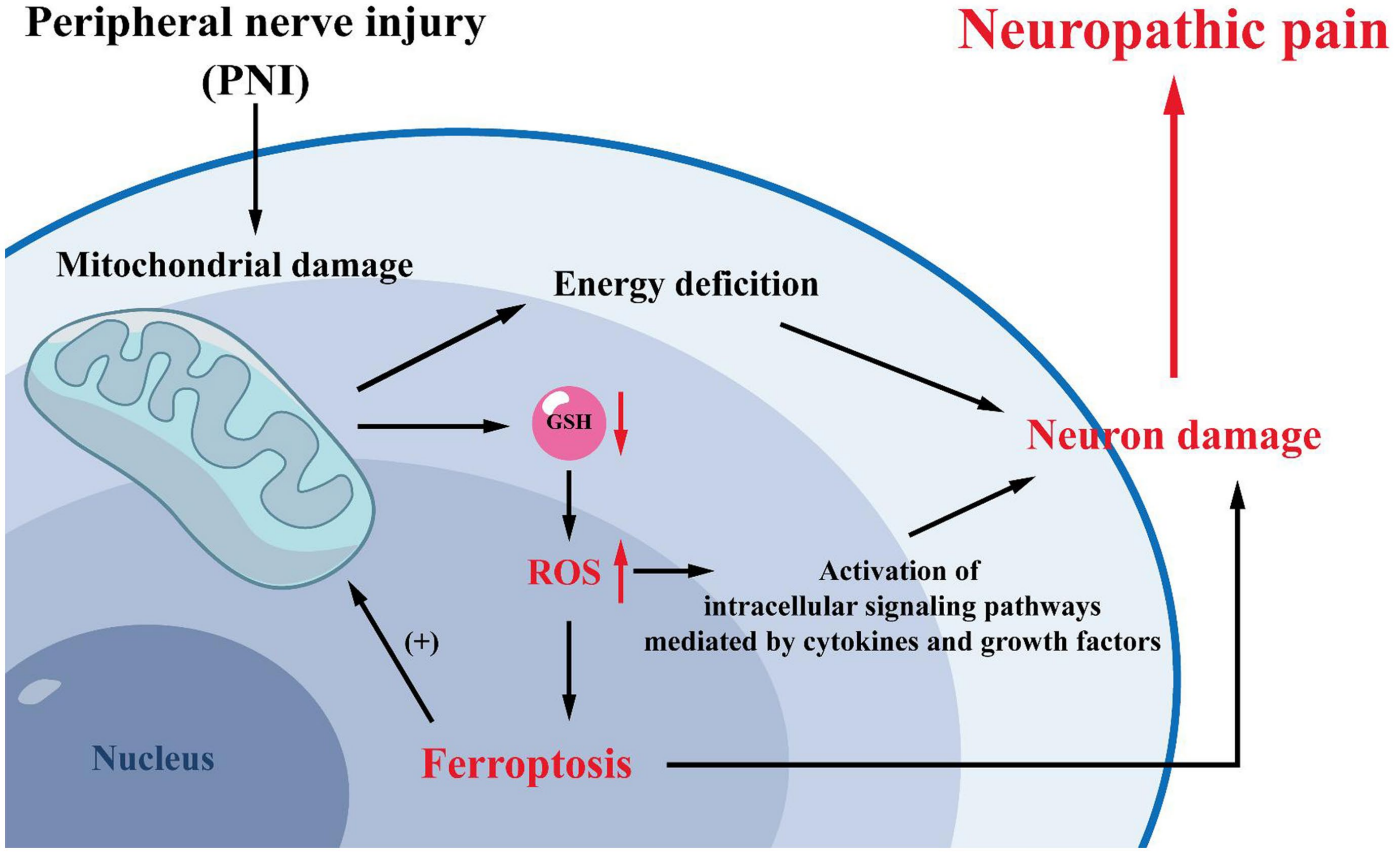
Mechanisms related to mitochondria. Mitochondrial damage is often observed after peripheral nerve injury. Mitochondrial damage will lead to increased intracellular GSH consumption and ROS production, promoting the development of ferroptosis. And ferroptosis can induce NP by reducing the activation of spinal cord dorsal horn neurons and astrocytes. Meanwhile, high levels of intracellular oxidative stress can activate cytokine- and growth factor-mediated intracellular signaling pathways, further leading to cells and tissue damage and exacerbating NP. Furthermore, mitochondria are the primary site of energy production in cells, and mitochondrial damage will result in inadequate intracellular energy supply, exacerbating neuronal damage and leading to NP.

Glutamine and cystine are two amino acids required for the synthesis of GSH, which may prevent iron prolapse caused by impaired lipid metabolism, including a decrease in ROS accumulation (Miess et al., 2018). The main way in which cysteine enters the cell as a raw material for glutathione synthesis is through xc^−^ system. Xc^−^ system is a heterodimeric amino acid transporter protein consisting of two subunits: solute carrier family 7 member 11(SLC7A11; also known as xCT) and solute carrier family 3 member 2 (SLC3A2, also known as CD98 or 4F2; Liu et al., 2021). It has been shown that secretion of INF-γ activates immune cells (e.g., CD8^+^ T cells), which downregulates the expression of xc^−^ system components to suppress cellular uptake of cystine, promoting ferroptosis induced by glutathione depletion (Zhong et al., 2022). GPX4 is the core enzyme regulating the glutathione system of the endogenous antioxidant system, NRF2 acts as a transcription factor to increase the expression of SLC7A11 and GPX4. GPX4 can increase peroxidase activity, reduce the toxicity of lipid peroxides, and maintain the cell membrane lipid bilayer stability, finally inhibit ferroptosis (Koppula et al., 2018).When xc^−^ system function is damaged or intracellular cysteine is inadequate, GSH synthesis decreases, causing ferroptosis. Glutamate is one of the most important amino acids in the body and plays a crucial role in the nervous system (Onaolapo and Onaolapo, 2021). Its reduction may be associated closely with intracellular cystine starvation, which results in ferroptosis (Figure 3).

### 3.2. Abnormal amino acid metabolism

Ferroptosis associated with abnormal amino acid metabolism is primarily related to abnormal glutathione metabolism. GPX4, xc^−^ system, sulfur transfer pathway, and a range of genes and regulators linked to GSH biosynthesis and degradation can be involved in iron sparing (Xu et al., 2021).

GSH is the central element of amino acid metabolism in ferroptosis and is made up of cysteine, glutamate, and glycine (Stockwell et al., 2017). In the cells, cystine is reduced to cysteine to participate in GSH synthesis (Zhang and Forman, 2012). GPX4 is a key enzyme for scavenging lipid oxygen radicals, using glutathione as a substrate (Chen et al., 2023). GPX4 overexpression inhibits ROS production and lipid peroxidation, while decreased GPX4 activity or expression results in the accumulation of intracellular lipid peroxides, contributing to ferroptosis (Mengstie et al., 2023; Poschel et al., 2023). GPX4, once activated, is capable of converting GSH to oxidized GSSG and reducing toxic lipid peroxides (L-OOH) to non-toxic lipid alcohols (L-OH), suggesting that GSH is an essential protective metabolite against ferroptosis (Hwang et al., 2021). RAS Selective lethal 3 (RSL3) is a ferroptosis inducer with an important role in GPX4-regulated ferroptosis (Wu et al., 2020). RSL3 can covalently bind to selenocysteine, the nucleophilic active site of GPX4, causing impaired GPX4 activity and thus provoking ferroptosis (Yang et al., 2016).

### 3.3. Abnormal lipid metabolism

Lipids are essential for the maintenance of cellular morphology and function, including biofilm composition, metabolism, energy storage, and signaling (Saleem et al., 2023). Lipid metabolism is also critical to the occurrence of ferroptosis. Reactive oxygen species are a group of partially reduced oxygen-containing molecules, including superoxide anion (O^2−^), hydrogen peroxide (H_2_O_2_), and hydroxyl radical (OH), which are critical for preserving the stability of cells and tissues (Li Y. et al., 2023). The accumulation of lipid ROS is a signature feature of the increased ferritin. It has been shown that polyunsaturated fatty acids (PUFAs) are important components of the lipid bilayer of cell membranes and are the molecular foundation for cell membrane fluidity (Hu H. et al., 2022). Ferroptosis is a mode of cell death caused by extra oxidation of phospholipids including PUFAs (Xu et al., 2021). Most ferroptosis-associated ROS can interact with PUFAs on lipid membranes to produce lipid ROS, and when large amounts of lipid ROS are accumulated in the cell, ferroptosis is expected to occur (Meng et al., 2022). Lipoxygenases (LOXs) may catalyze the oxidation of PUFAs to lipid peroxides, thus participating in the formation of iron-dependent lipid ROS. Under the presence of large amounts of iron ions in the cytoplasm, LOXs can catalyze the formation of toxic lipid radicals from PUFAs, ultimately contributing to cellular damage and facilitating ferroptosis (Kagan et al., 2017). At the same time, protons near PUFA may be transferred by these poisonous lipid radicals, driving a new circle of lipid oxidation reactions, and ultimately resulting in more aggressive oxidative damage (Yang et al., 2016).

Two enzymes play a key role in the synthesis of PUFAs during the onset of iron death: acyl-coenzyme A synthase long-chain family member 4 (ACSL4) and lysophosphatidylcholine acyltransferase 3 (LPCAT3; Xue et al., 2023). When these two genes were knocked out, PUFA synthesis was reduced, causing the inhibition of ferroptosis. ACSL4 is a member of the ACSL family, which catalyzes the formation of acyl coenzymes from fatty acids (Ma et al., 2021). ACSL4 overexpression facilitates GPX4 inhibitor-induced ferroptosis. On the contrary, the knockdown of ACSL4 can prevent GPX4 depletion-induced ferroptosis. High LPCAT3 expression upregulates the level of unsaturated fatty acids in biofilms and increases the sensitivity of cells toward ferroptosis. The expression levels of ASCL4 and LPCAT3 are now used as indicators to detect the sensitivity of ferroptosis sensitivity.

### 3.4. Abnormal iron metabolism

Numerous aspects of iron metabolism, such as iron uptake, storage, and utilization, have major roles in the modulation of ferroptosis (Li J. et al., 2023). Under normal physiological conditions, intracellular iron uptake and metabolism are in a dynamic equilibrium. Iron in food is absorbed by intestinal epithelial cells mainly in the form of Nonheme iron (Fe^3+^). After binding to transferrin (TF), Fe^3+^ enters the cell through the transferrin receptor (TFR) on the membrane. Subsequently, Fe^3+^ is reduced to Fe^2+^ in cells via the six-transmembrane epithelial antigen of prostaglandin 3 (STEAP3), which is then released into the cytoplasmic unstable iron pool via divalent metal transporter protein 1 (DMT1) or zinc-iron regulatory protein family 8/14 (ZIP8/14) or stored as ferritin (Frazer and Anderson, 2014). The abnormal expression or dysfunction of these involved proteins leads to an unbalance of intracellular iron ion concentration, resulting in iron overload. Excessive intracellular Fe^2+^ will generate excessive ROS through the Fenton reaction, leading to a continuous accumulation of intracellular ROS and triggering ferroptosis (Zhao et al., 2023).

### 3.5. Sulfur transfer pathway

Cysteine, an essential non-essential amino acid in cell proliferation, is not only an important raw material for protein synthesis but also a key substrate for the synthesis of glutathione antioxidants. More than 40% of cysteine in mammals is obtained from food (Mou et al., 2019). Cysteine can enter the cell through two major pathways: one way is dependent on the xc- system and the other on the sulfur transfer pathway. When cysteine intake is insufficient, homocysteine will combine with serine in the body, thus entering the sulfur transfer pathway and converting to cysteine precursor and entering the cysteine pool (Yang et al., 2022). Injury in both of these pathways is known to contribute to the development of ferroptosis.

## 4. Ferroptosis and cells in central nervous system

In recent years, an increasing number of research focused on the developmental mechanisms of ferroptosis at different levels of the sensory system and in various types of cells, while also focusing on exploring the potential linkages embedded therein. Nowadays, what has been studied more clearly are the neurons and glial cells of the central nervous system.

In astrocytes, high expression of molecules associated with ferroptosis, such as SLC7A11, AQP1, AQP4, AQP9, pannexin-1, and P2X7 receptor transcripts, could be observed (Ottestad-Hansen et al., 2018; Komatsu et al., 2022). Ferroptosis in astrocytes may be induced by the angiotensin II (Ang II)-activated or Brain-derived neurotrophic factor (BDNF)-activated Nrf2/heme oxygenase-1 signaling pathway (Ishii et al., 2019; Li et al., 2021). Some other downstream genes regulated by Nrf2 may be related to cell-to-cell interactions, and the close cooperation and interactions between astrocytes, oligodendrocytes, microglia, and neurons are the basis for maintaining the function of the central nervous system and the normal life activities of living organisms.

In oligodendrocytes, the amount of iron is 20 times higher than in astrocytes, and since iron is a cofactor required for myelin synthesis, an excessive amount of intracellular iron will contribute to the development of ferroptosis (Thorburne and Juurlink, 2002; Van Rensburg et al., 2019; She et al., 2021). Also, the investigators observed that oligodendrocyte precursor cells (OPCs) are more easily susceptible to the effects of extracellular free iron and intracellular cysteine depletion, which leads to an increase in lipid peroxidation, a blockage of oligodendrocyte differentiation, and cell death.

In microglia, which is a type of scavenger cell, changes in iron content may affect the survival of oligodendrocytes (Zhang et al., 2006). After spinal cord injury, microglia activation will lead to further motor cortical iron overload, triggering motor neuron ferroptosis and preventing the recovery of motor function (Hu W. et al., 2022).

We strongly believe that with further investigations of the above cells, as well as Schwann cells and satellite glial cells in the peripheral nervous system, we will soon be able to clarify the essential role of ferroptosis in nerve injury and its progression and to develop emerging therapeutic avenues for neurological disorders by targeting and regulating the pathways of ferroptosis at different levels of the sensory system and in different types of cells.

## 5. The relationship between neuropathic pain and ferroptosis

Recently, scholars have found a correlation between ferroptosis and the development of neurological diseases such as PD and AD (Lei et al., 2020). It is involved in the pathogenesis of neurological-related diseases through the regulation of intracellular iron ion levels, glutamate excitability, and the occurrence of oxidative stress. Studies have shown that iron accumulation and lipid peroxidation are associated with the development of various neurological diseases, accompanied with reduction of GSH and GPX4 levels (Wu et al., 2018). AD is the most common neurodegenerative disease. Iron levels are significantly elevated in the severely damaged hippocampus in AD patients (Lou et al., 2021). Iron accumulation in brain tissue induces a significant production of ROS in brain cells, which ultimately leads to oxidative damage (Feng et al., 2022). PD is mainly due to the degeneration of dopaminergic neurons (Liu et al., 2020). The abundance of iron in neurons is associated with ferroptosis. Amyotrophic lateral sclerosis (ALS) is a neurodegenerative disease affecting motor neurons in the cerebral cortex, spinal cord, and brainstem. A large accumulation of iron in the spinal cord can be detected in the lesioned areas of ALS (Li et al., 2020a). Moreover, ALS patients have increased levels of lipid peroxidation in erythrocytes and decreased GSH levels, which in turn exacerbate the degeneration of ALS motor neurons (Johnson et al., 2012). Related studies have shown that inhibitors of ferroptosis have significant therapeutic effects in neurological-related diseases such as PD and spinal cord injury (Southon et al., 2020). These findings provide evidence that neurodegenerative diseases are closely related to ferroptosis. Neuropathic pain, a typical neurodegenerative disease, is also inextricably linked to ferroptosis (Wang et al., 2021). Understanding the relationship between NP and ferroptosis provides promising ideas for the treatment of NP.

**Figure 4:**
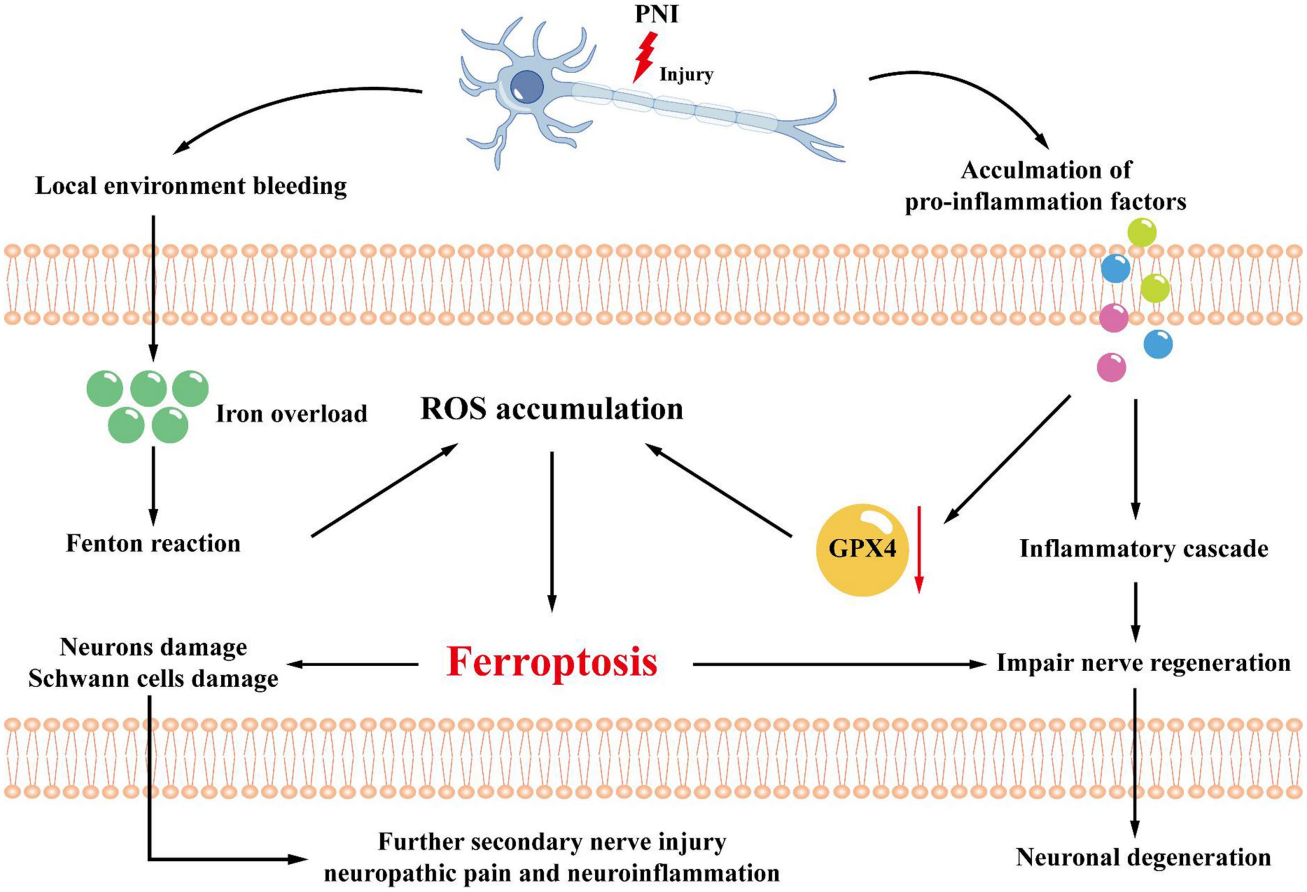
Mechanisms related to the microenvironment. After peripheral nerve injury, local environmental bleeding leads to local iron ion overload, increasing the Fenton reaction, adding ROS accumulation, and inducing ferroptosis. At the same time, the microenvironment also releases pro-inflammatory factors, which can activate GPX4 and promote ferroptosis through the inflammatory cascade. Ferroptosis will lead to further damage to Schwann cells and axons, accelerating the progression of nerve damage and leading to secondary nerve injury, neuropathic pain, or neuroinflammation.

Neuropathic pain is chronic, intractable, and not relieved by analgesics. One study found a decrease in GPX4 levels and an increase in ACSL4 after CCI injury. Electron transmission microscopy showed characteristic changes of ferroptosis in the mitochondria of cells at the dorsal horn of the spinal cord such as contraction of mitochondria, increase in the density of the inner mitochondrial membrane, decrease in the mitochondrial cristae, and rupture of the outer mitochondrial membrane. In addition, the iron content, ROS, and MDA in the spinal cord of CCI model rats were elevated, while GSH was decreased. Among them, MDA can react with various amino acids to reduce cell activity and even promote apoptosis (Jiang et al., 2023). It indicates that iron accumulation as well as oxidative stress were aggravated in the CCI model. In addition, Fer-1, a ferroptosis inhibitor, upregulates the expression of GPX4 and downregulates the expression of ACSL4 (Wang et al., 2023). And in a related study, it was found that treatment with ferrostatin-1 significantly inhibited the onset of oxidative stress in brain tissue, improved the structure of brain tissue after hemorrhage, protected neurons, and promoted neurological function in recovery of rats. Another specific inhibitor, liproxstatin-1 (Lip-1), also significantly inhibited the elevated levels of ROS induced by mitochondrial lipid peroxidation and restored GPX4 expression levels, which in turn effectively reduced chronic compression injury of the rat (Song et al., 2023). A recent study showed that CCI-induced mechanical and thermal nociceptive abnormalities in rats were significantly alleviated by intraperitoneal injection of Lip-1, while a decrease in iron content and a reduction in lipid peroxidation responses in spinal cord tissue were observed, along with a return to normal GPX4 expression levels. Based on this study, we further found that a single intrathecal injection of Lip-1 provided significant pain relief in a dose-dependent manner. Continuous intrathecal injection of Lip-1 effectively reversed mechanical nociceptive abnormalities in rats without tolerance. There was also a study in which Lip-1 was injected intrathecally for 7 consecutive days. The results showed that MWT in rats was statistically significantly decreased only at 7d compared with the control group. This result suggests that early intrathecal injection of Lip-1 can delay, but not completely prevent, the onset of pain. In addition, western blot results showed that the expression level of GPX4 in the spinal cord of painful rats was significantly downregulated. And multiple intrathecal injections of Lip-1 significantly reversed this effect. Immunodouble-labeling techniques revealed that GPX4 was expressed on neurons throughout the dorsal horn of the spinal cord, but very rarely co-expressed with astrocytes and microglia. These results suggest that ferroptosis is involved in the development of pain. However, the therapeutic dose of ferroptosis inhibitor and the optimal effective dose concentration for treating NP has to be further determined by setting up a gradient concentration group of ferroptosis inhibitors. The opposite role is played by the ferroptosis inducer erastin, which has the advantage of being highly effective, rapid, and longer acting than other iron death inducers. It can induce ferroptosis in cells through various actions such as inhibition of the cystine-glutamate transport pathway, activation of the p53 gene pathway, and elimination of the inhibitory pathway of microtubulin on voltage-dependent anion channel (VDAC; Sinha et al., 2023). RSL3 is a selective agonist of ferroptosis. In pain studies, it was shown that intrathecal injection of the ferroptosis agonist RSL3 resulted in a significant decrease in pain thresholds and caused mechanical nociceptive abnormalities in normal rats. In summary, ferroptosis is involved in NP development and maintenance by blocking the activity of neurons and astrocytes in the dorsal horn of the spinal cord (Wang et al., 2021; Table 1). However, whether the occurrence of ferroptosis is involved in changes in the inflammatory microenvironment of the NP requires further relevant studies (Figure 4).

**Table 1:** Model, mechanism and for targets for ferroptosis in neuropathic pain.

Model	Regent	Key mechanisms	Targets
CCI	Fer-1 (inhibitor) erastin (inducer)	blocking neuron and astrocyte activation in the spinal dorsal horn	GPX4↓ ACSL4↑ Fe↑
CCI	Lip-1 (inhibitor)	peripheral nerve injury	GPX4↓, Fe↑, ACSL4↑, ROS↑
CCI	DEmRNAs DElncRNA	Using RNA-Seq, induce memory impairment in response to chronic pain stress	GPX4↓, ACSL4↑ SLC7A11, SLC1A5, PTGS2, Fe↑, ROS↑
CP/CPPS	DFO/ EDA	inhibit oxidative stress, ferroptosis, inflammation, fibrosis, mast cell activation	ROS↑, MDA↑, SOD↓, CAT↓, Xc(−)/GPX4, ACSL4/LPCAT3, NRF2/HO-1↓
Chronic pelvic pain	N-cetylcysteine, curcumin, melatonin, vitamin C and E	neurogenic inflammation, macrophages, pro-inflammatory cytokines, pain-inducing prostaglandins	Fe↑, ROS↑
SNI	SIRT2 FPN1	inhibit iron accumulation, reduced oxidant stress, reversing ACSL4 and GPX4	GPX4↓, ACSL4↑, Fe↑
SNL	GSEA GSVA	NK cells, RGS4, CXCL2, DRD4 and other genes related to ferroptosis	RGS, HIF-1
GCH1-KD cells	GCH1	play a pivotal role in microglial activation, which is essential for NP.	miR-1a-3p, miR-133a-3, miR-7a-5p,miR-10a-5p
CFA	GAS	exhibits potential neuroprotective effects through inhibition of ferroptosis	GPX4↑, FTHI↑, HO-1↑, PTGS2↓
CFA	Lip-1 inhibitor	ferroptosis in the spinal cord and DRG induce inflammatory pain	GPX4↓, ACSL4↑, Fe↑, ROS↑
OA	–	DNA methylation, histone modification, and microRNA regulation, and mediators	Fe↑, ROS↑
OA	DEFRGs	inhibited ferroptosis and cartilage degeneration	SLC3A2
IVDD	–	decreasing viability and increasing extracellular matrix degradation of nucleus pulposus cells, annulus fibrosus cells, or endplate chondrocytes.	Fe↑, ROS↑
IVDD	–	RNA modulations through RNA interference and regulation of non-coding RNAs,	Fe↑, ROS↑
IVDD	ATF3	ROS production, inflammatory response, and ECM degradation	Fe↑, ROS↑

## 6. New methods for neuropathic pain treatment based on ferroptosis

In recent years, as the relationship between ferroptosis and NP has been more widely uncovered, researchers have focused increasingly on finding new approaches to NP treatment based on ferroptosis, aiming to explore more potential targets for NP therapy.

The Sirtuin (SIRT) family of proteins are conserved proteins classified as class III histone deacetylases (Wu et al., 2022). SIRT2 is the only sirtuin that is mainly found in cytoplasmic (Wang et al., 2019). Ferroportin 1 (FPN1) is a transmembrane protein that translocates iron ions and has an essential role in maintaining cellular homeostasis. For the first time, a recent study suggested that SIRT2 may alleviate NP by inhibiting ferroptosis in SNI rats. The investigators showed that both SIRT2 and FPN1 expression was reduced in microglia in the dorsal horn of the spinal cord of SNI rats, while the intrathecal injection of SIRT2 overexpressed recombinant adenovirus (Ad-SIRT2) upregulated the expression of SIRT2 and FPN1 in the spinal cord, decreased the accumulation of iron, and attenuated mechanically abnormal hyperalgesia in the SNI rats (Zhang X. et al., 2022).

In addition to the above, some other studies have shown that intraperitoneal injection of liproxstatin-1, a specific ferroptosis inhibitor, attenuates mechanical and thermal hypersensitivity in CCI rats, as well as reducing NP induced by peripheral nerve injury in rats (Guo et al., 2021). Overexpression of NADPH oxidase 4 (Nox4) in DRG can cause ferroptosis and lead to NP. Methyl ferulic acid (MFA) given by gavage can alleviate the NP symptoms in SNI rats by inhibiting the increase of Nox4 in DRG (Liu et al., 2023).

These investigations have provided new insights into the treatment of NP, however, therapies targeting ferroptosis have not yet been applied to the clinical treatment of NP, although significant results have been demonstrated in the SNI and CCI rat models. We believe that in the near future, with the further exploration of the relationship between ferroptosis and NP, more efficient new methods for the clinical treatment of NP can be exploited.

## 7. Conclusion

The mechanism of NP is complex, involving multiple cells, molecules, and loops. From the regulatory mechanism of ferroptosis, it can be seen that ferroptosis can be induced by increasing iron ion levels, glutamate levels, phospholipids, and polyunsaturated fatty acid levels, or by removing GSH, GPX4, NADPH, lipid antioxidants. Ferroptosis is widely involved in the physiopathological processes of the body and has an important role in maintaining cellular tissue homeostasis. Elevated intracellular ROS levels in spinal cord dorsal neurons induce the activation of the neuronal ferroptosis pathway and participate in the process of NP. Exploring the relationship between ferroptosis and NP provides a new theory for NP which may be a new approach and target for its treatment.

## Author contributions

GH drafted the manuscript, checked and edited the content, and format of this manuscript before submission. LL and GH conceived the idea of this review. RG and CH performed literature searching and co-wrote this paper. MY, LG, and QF performed critical editing and participated in the constructive outline, discussions, and editing. XQ and GS conducted language editing and re-checking literature. LG was responsible for all the revision work, revised the references, adjusted the structure of the article, redrew Figure 2, and supplemented Figures 3, 4. All authors contributed to the article and approved the submitted version.

## Abbreviations

CCI: chronic contractile injury
SNI: spared nerve injury
Fer-1: ferrostatin-1
Lip-1: liproxastin-1
GSH: glutathione
GPX4: glutathione peroxidase 4
ACSL4: acyl-CoA synthetase long-chain family member 4
SLC7A11: solute carrier family 7 member 11
ROS: reactive oxygen species
MDA: malondialdehyde
SOD: superoxide dismutase
DEmRNAs: differentially expressed mRNAs
DElncRNAs: differentially expressed long noncoding RNAs
CP/CPPS: Chronic prostatitis/chronic pelvic pain syndrome
Nrf2: nuclear factor erythroid 2-related factor 2
GAS: gastrodin
ECM: extracellular matrix
ssGSEA: single-sample gene set enrichment analysis
GCH1: GTP cyclohydrolase 1
SIRT2: sirtuin 2
DRG: dorsal root ganglion
FPN1: ferroportin 1
LDH: lactate dehydrogenase
CFA: complete Freund’s adjuvant
DFO: Iron chelator deferoxamine
EDA: free radical scavenger edaravone
OA: Osteoarthritis
DEFRGs: differentially expressed ferroptosis-related genes
IVDD: Intervertebral disc degeneration
